# Effects of high-volume hemofiltration on lungoxygenation in patients with septic shock

**DOI:** 10.1186/2197-425X-3-S1-A58

**Published:** 2015-10-01

**Authors:** EA Tishkov, AN Kuzovlev, OB Bukaev, YV Luzganov

**Affiliations:** Moscow University of Medicine and Dentistry, Dept. of Anesthesiology and Reanimatology, Moscow, Russian Federation; V.A. Negovsky Research Institute of General Reanimatology, Moscow, Russian Federation; Moscow City Hospital #1, Moscow, Russian Federation

## Introduction

High-volume hemofiltration (HVHF) is technically possible in severe acute disease abdominal cavity patients complicated with multiple organ dysfunction syndrome (MODS). Continuous HVHF is expected to become a beneficial adjunct therapy for acute pancreatitis and peritonitis complicated with MODS. In this study, we aimed to explore the effects of fluid resuscitation and HVHF on alveolar-arterial oxygen exchange, the Acute Physiology and Chronic Health Evaluation II (APACHE II) score in patients with septic shock.

## Objectives

This study was undertaken to explore the effects of fluid resuscitation and HVHF on alveolar-arterial oxygen exchange, APACHE II score in patients with septic shock.

## Methods

A total of 45 septic shock patients, who were admitted to ICU, were enrolled in this retrospective study. The patients were randomly divided into two groups: fluid resuscitation (group A, n = 20), and fluid resuscitation plus high-volume hemofiltration (group B, n = 25). The levels of O2 content of central venous blood (CvO2), arterial oxygen content (CaO2), alveolar-arterial oxygen pressure difference P(A-a)DO2, ratio of arterial oxygen pressure/alveolar oxygen pressure (PaO2/PAO2), respiratory index and oxygenation index were determined. The oxygen exchange levels of the two groups were examined based on the arterial blood gas analysis at different times (0, 24, 48 hours and 5 days of treatment) in the two groups. The APACHE II score was calculated before and after 5-day treatment in the two groups.

## Results

The levels of CvO2, CaO2 on day 5 in group A were significantly lower than those in group B (CvO2: 0.62 ± 0.22 vs. 0.74 ± 0.24, P < 0.05; CaO2: 0.82 ± 0.36 vs. 0.95 ± 0.42, P < 0.05). The level of oxygen extraction rate (O2ER) in group A on the 5th day was significantly higher than that in group B (29.7 ± 2.5 vs. 22.7 ± 3.5, P < 0.01). The levels of P(A-a)DO2 and respiratory index in group B on the 5th day were significantly lower than those in group A. The levels of PaO2/PAO2 and oxygenation index in group B on 5th day were significantly higher than those in group A (P < 0.05 or P < 0.01). The APACHE II score in the two groups reduced gradually after 5-day treatment, and the APACHE II score on the 5th day in group B was significantly lower than that in group A (8.0 ± 2.8 vs. 18.2 ± 5.8, P < 0.01).

## Conclusions

HVHF combined with fluid resuscitation can improve alveolar-arterial-oxygen exchange, decrease the APACHE II score in patients with septic shock, and thus it increases the survival rate of patients.

